# A Simple Strategy to Mitigate the Aliasing Effect in X-band Marine Radar Data: Numerical Results for a 2D Case

**DOI:** 10.3390/s110101009

**Published:** 2011-01-18

**Authors:** Francesco Serafino, Claudio Lugni, Josè Carlos Nieto Borge, Francesco Soldovieri

**Affiliations:** 1 Institute for Electromagnetic Sensing of the Environment, National Research Council, Via Diocleziano 328, Napoli I-80124, Italy; E-Mail: serafino.f@irea.cnr.it; 2 INSEAN, the Italian Ship Model Basin, Department of Seakeeping and Maneuverability, Via di Vallerano 139, Roma I-00128, Italy; E-Mail: c.lugni@insean.it; 3 Department of Signal Theory and Communications, University of Alcala, Alcala de Henares 28805, Spain; E-Mail: josecarlos.nieto@uah.es

**Keywords:** X-band radar, aliasing problem, reconstruction approach

## Abstract

For moderate and high speed values of the sea surface current, an aliasing phenomenon, due to an under-sampling in the time-domain, can strongly affect the reconstruction of the sea surface elevation derived from X-band radar images. Here, we propose a de-aliasing strategy that exploits the physical information provided by the dispersion law for gravity waves. In particular, we utilize simplifying hypotheses and numerical tests with synthetic data are presented to demonstrate the effectiveness of the presented method.

## 1. Introduction

It is well known that images collected by nautical X-band radar enclose sea state information [1–3]. In particular, the space-time evolution of the sea surface elevation can be reconstructed from the X-band radar images arising by the backscattering phenomena of the electromagnetic fields with the sea surface ripples (e.g., capillary waves) in the microwave regime [4,5]. In fact, due to the modulation effects induced on the signal backscattered by the capillary waves, the longer surface gravity waves may be visible by the radar. The echo received by the radar undergoes to a modulation due to the change of the angle under which the sea wave is viewed (tilt modulation) and to the electromagnetic shadowing of the sea surface induced by the higher waves preceding the wave under investigation. These modulation effects introduce a non linear spectral distortion, mainly at high wave number [1,2].

Therefore, data processing is necessary to estimate the sea-wave spectrum starting from the spectrum of the radar image (image spectrum). In particular, data processing is formulated as a linear inverse problem where, starting from the radar images sequence, the sea surface elevation is obtained as a function of two spatial variables (covering to the zone investigated by the radar) and of the time. The linear inversion scheme [1–3,6] can be summarized as follows: after a Fourier Transform of the time-space data, a spectral filter is used to reduce the effect of all the undesired phenomenon via a dispersion relation [1,6]. Furthermore, the use of the Modulation Transfer Function (MTF) allows the passage from the image (radar) spectrum to the wave spectrum [7,8]; finally, the resulting spectrum is Fourier anti-transformed to return to the space-time domain.

Being the inversion procedure strongly based on Fourier transform techniques, an aliasing phenomenon may arise in presence of an inappropriate time steps adopted to sample the radar image. In fact, due to the slow repetition time *Δt* (*Δt* is of order 1–2 s) of a nautical radar, it arises that the waves can be temporally under-sampled (less than twice each period, *i.e.*, the Nyquist frequency is exceeded) and the aliasing effect occurs [9–11].

In this paper, by considering the simpler case of a 2D problem, *i.e.*, the sea surface elevation is a function of only one spatial variable (denoted by *x*) and of the time, first we analyze how the under-sampling in time domain affects the reconstruction procedure in both the cases of presence and absence of a surface current moving in the same direction of the waves (head waves). Here, we mean the surface current as given by two contributions: the first one is the “physical” sea surface current; the second one is an “equivalent” sea surface current that arises due to the relative movement of the radar system with respect to the sea surface.

Therefore, the problem at hand is relevant to the naval context, where the characterization of the sea state with X-band radar system becomes a challenge, when a semi-displacement or a planning vessel is considered, even with head sea conditions. The high speed of the ship emphasizes the aliasing phenomenon of the data in the time domain, making it necessary to tackle the problem.

The aliasing problem has been already addressed in [9–12], where a strategy was also proposed to solve this problem. In particular, the reconstruction procedure is based on the accurate knowledge and exploitation of the gravity dispersion relation and aims at reconstructing the signal also when the frequency of the spectral energy exceeds the Nyquist frequency. This is performed thanks to the application of spectral symmetries [10] and the sampling theorem is not violated because the dispersion relation as additional physical information is used for the unambiguous reconstruction of the spectrum [11].

As general comment, it is worth noting that, here, de-aliasing is possible since we exploit information on the physical model of the phenomenon and therefore the case at hand is completely different from the classical one reported in the text books [13] where, in absence of any *a priori* and model information, it is not possible to estimate the aliasing and neither apply any mitigation strategy. As a matter of fact, de-aliasing techniques have been already applied in other applications as video images processing [14,15], optical imaging [16].

In this paper, we move in the same framework of [10–12] and we present a strategy to mitigate the effect of the aliasing phenomenon, which accounts for the gravity waves dispersion relation but in a different way compared to the strategy presented in [10–12]. In particular, differently from the procedure (exploiting spectral symmetries) presented in [10–12], here the folding problem is mitigated thanks to a procedure where the exploitation of a “virtual surface current” permits one to go beyond Nyquist’s limit and reconstruct the aliased dispersion shell. More specifically, the reconstruction of the aliased dispersion shells is obtained by simply exploiting a variable change based on the gravity waves dispersion relation. The effectiveness of the proposed strategy is shown with results against synthetic clutter and clutter-removed data and also a comparison is presented with the strategy outlined in [12].

Therefore, the paper is organized as follows. Section 2 reports briefly the data processing approach for sea state monitoring from X-band radar data. In Section 3, the aliasing problem is sketched by referring to head waves whereas a strategy to address this problem is proposed in Section 4. Reconstruction results against synthetic data are shown in Section 5 and finally, the Conclusions follow.

## 2. The Data Processing Approach

**Figure 1.**
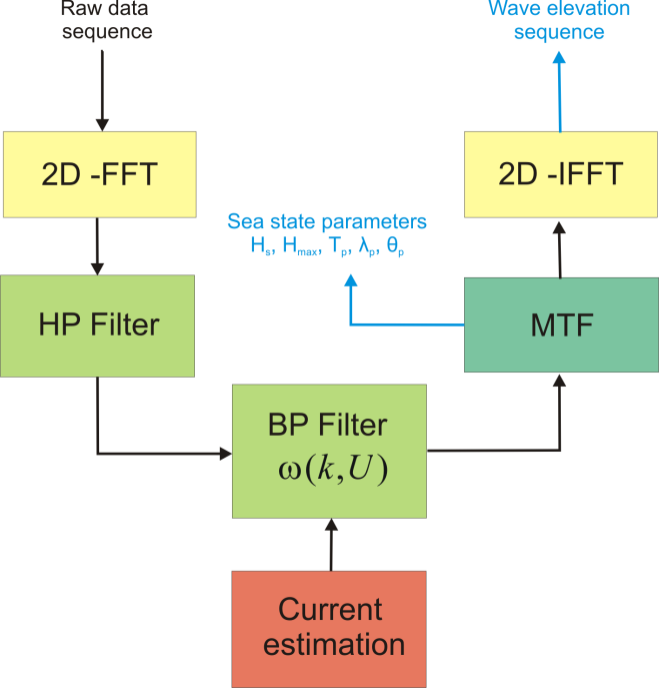
Block diagram of the inversion procedure.

This Section briefly presents the scheme commonly exploited to reconstruct the time-spatial evolution of the sea surface elevation starting from the X-band radar images. For sake of simplicity, the inversion approach is presented in the 2D case with a sea surface elevation that is considered as a function of the time *t* and of only the spatial variable *x*. The proposed method can be generalized to the 3D full radar scene considering the spatial variables *x* and *y*. The reconstruction procedure is schematized in the block diagram of Figure 1 where each block is detailed below.

The initial step consists in Fourier transforming (2D Fast Fourier Transform, 2D-FFT) the raw data images sequence so to obtain the 2D image spectrum *F*(*k,ω*). A High-Pass (HP) filter is then applied to the image spectrum in order to remove the effects due to the received signal power decay along the spatial range (*x*-variable) [2].

In the second step the expected linear gravity wave components are extracted from the HP filtered image spectrum *F_I_*(*k,ω*). More in detail, the dispersion relation of linear gravity waves, which relates the wave number *k* to the pulsation *ω(k*) and the sea surface current *U*, is considered:

(1) $$\omega (k)=\sqrt{g\left|k\right|\;\text{tanh}(\mathrm{kh})}+\mathrm{kU}$$

where *g* is the gravity constant and *h* the water depth [6]. In the following we assume infinite depth conditions, *i.e.*, tanh(*kh*) ≅ 1.

In order to properly apply the filtering process based on the dispersion relation, an accurate estimation of the current value *U* is necessary. The current determination represents one of the key steps in the full inversion procedure; in fact, a bad estimate of *U* arises an incorrect filtering of the wave components. Several strategies can be adopted to the purpose, like the ones proposed in [17–19].

Once the current *U* has been estimated, the band-pass (BP) filter *G*(*k*,*ω*,*U*) is built according to Equation (1) and applied to the image spectrum *F_I_*(*k,ω*) (see block diagram of Figure 1). As a result of the filtering procedure, the spectrum *F̃*(*k*,*ω*) is obtained [18,19]. The minimization of the radar modulation effect introduced in the sea wave backscattered signal, allows to get the expected sea-wave spectrum *F_W_*(*k,ω*) starting from the filtered image spectrum *F̃_I_*(*k,ω*). In particular the Modulation Transfer Function (MTF) |*M*(*k*)|^2^ is applied to the filtered spectrum *F̃_I_*(*k,ω*) according to:

(2) $$F_{W}\;\left(k,\;\omega \right)=\frac{{\tilde{F}}_{I}\;\left(k,\;\omega \right)}{{\left|M\;(k)\right|}^{2}}$$

being |*M*(*k*)|^2^ = *k^β^*; empirical analysis provided *β =* −1.2 as a good estimation [2,7,8].

Finally, the knowledge of the sea wave spectrum *F_W_*(*k,ω*) allows the calculation of the typical sea state parameters and the time-space evolution of the sea surface elevation *η*(*x*, *t*) can be recovered by performing an inverse 2D-FFT on the function *F_W_*(*k,ω*).

## 3. The Effect of the Aliasing

This Section is devoted to investigating the effect of the aliasing phenomenon on the performance of the inversion procedure described in the Section 2. This analysis provides the basis to propose an effective de-aliasing strategy that will be presented in the Section 4. Let us first consider the case of absence of sea surface current, *i.e.*, *U* = 0; in this case, the gravity waves dispersion relation becomes:

(3) $$\omega (k)=\sqrt{\left|k\right|g}$$

By defining *k_B_* as the maximum wave-number for which the spectrum of the radar image *F*(*k,ω*) can be assumed different from zero (in the test cases considered in this paper, we assume the spectrum is assumed to be different from zero when its modulus is larger than 0.1 times the peak value), Equation (3) provides the bandwidth *ω_B_* associated to *k_B_* as $\omega_{B}=\sqrt{k_{B}\;g}$. Apart from the modulation effects introduced by the specific radar sensing mechanisms [2,8], the radar image is a sampled version of the sea surface elevation *η*(*x*, *t*), collected at uniform sampling steps Δ*t* and Δ*x*. By defining $\omega_{s}=\frac{2\pi }{\Delta t}$ as sampling frequency and $k_{s}=\frac{2\pi }{\Delta x}$ as the spatial sampling frequency, and according to the well known Nyquist’s law [13], the reconstructed wave elevation *η*(*x*, *t*) will be not affected by aliasing if:

(4) $$\omega_{s}\geq 2\omega_{B}$$

and:

(5) $$k_{s}\geq 2k_{B}$$

For typical X-band radar images, the spatial step Δ*x* is suitable to follow the marine waves; therefore in our analysis we assume condition (5) to be satisfied and in the following only the under-sampling in the time domain will be considered.

**Figure 2.**
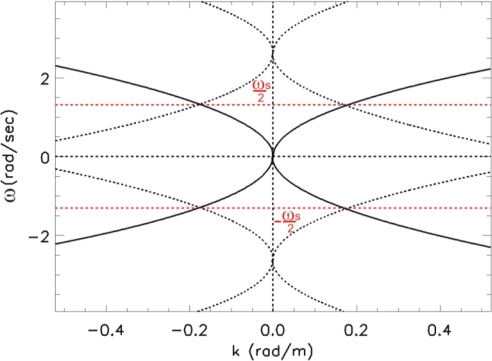
Folded spectrum related to the parameters of Table 1. The dotted lines depict the replicas of the spectrum.

When relation (4) does not hold, the replicas of the dispersion relation centered at ±*ω_s_* have a part falling within the allowable band $\left[-\frac{\omega_{s}}{2},\;\frac{\omega_{s}}{2}\right]$. This phenomenon is clearly indicated in Figure 2, where the aliasing of the spectrum is caused by the non proper choice of the measurement parameters as given in Table 1.

In particular, the undesired folding phenomenon occurs for the range of the wave-number *k_m_* ≤|*k*|≤*k_B_*, where *k_m_* (see Figure 2), is determined through:

(6) $$\omega_{m}=\omega_{s}/2=\frac{\pi }{\Delta t}=\sqrt{k_{m}\;g}$$

that provides:

(7) $$\lambda_{m}=\frac{2\pi }{k_{m}}=\frac{2{\Delta t}^{2}}{\pi }\;g$$

According to Equation (7), all the waves with a wavelength *λ_B_* *≤λ≤λ_m_* will be affected by the aliasing phenomenon. For sake of simplicity, only progressive waves, *i.e.*, waves whose spectrum has support in the first and third quadrant of the spectral plane (*ω/k*), are considered hereinafter. This entails that the phase velocity *ω/k* of these waves is positive, *i.e.*, they propagate along the positive *x*-axis. In this specific case, the folding phenomenon arises two undesired shells in the second and fourth quadrant of the spectral domain (*ω/k*) (see Figure 2). These folded (aliased) shells are characterized by a negative phase velocity and accordingly they are representative of “wrong” (with not physical meaning) waves propagating in the direction opposite to the true ones (see Figure 2).

**Figure 3.**
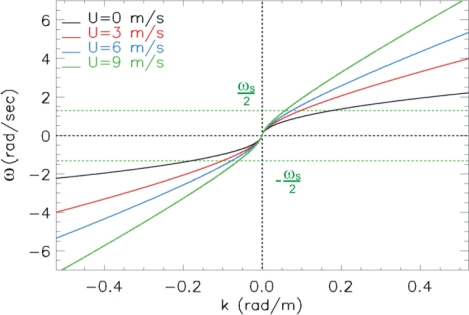
Dispersion relation for the progressive waves when the surface current *U* changes.

Let us turn now to consider the case of a non null sea surface current. In particular, we consider a surface current moving in the same direction of the waves (head waves). In this case, we have to consider the dispersion law (see Figure 3):

(8) $$\omega (k)=\sqrt{\left|k\right|g}+\mathrm{kU}$$

with *kU* ≥ 0. Figure 3 depicts the dispersion relation (8) at increasing positive values of the surface current *kU*; this Figure allows us to point it how the condition (4), stating the absence of aliasing, is more and more difficult to be satisfied as long as the surface current value *U* increases. Therefore, we can state that the presence of such a current emphasizes the effect of the aliasing by amplifying the folding phenomenon.

In fact, the bandwidth *ω_BP_*, corresponding to the maximum value of the wave-number *k_B_*, becomes:

(9) $$\omega_{\mathrm{BP}}\;\left(k_{B}\right)=\sqrt{\left|k_{B}\right|g}+k_{B}U=\omega_{B}+k_{B}U=\omega_{B}+\frac{\omega_{B}^{2}}{g}U$$

and relation (9) permits to determine the minimum value *Û* of the surface current *U* for which the spectrum folding arises. In fact, the folding problem arises when Equation (4) does not hold and this gives:

(10) $$\omega_{s}\leq 2\omega_{\mathrm{BP}}=2\left(\omega_{B}+\frac{\omega_{B}^{2}}{g}\hat{U}\right)$$

that after simple manipulations provides:

(11) $$\hat{U}\geq \left(\frac{\omega_{s}}{2}-\omega_{B}\right)\;\frac{g}{\omega_{B}^{2}}=\frac{\omega_{s}}{2k_{B}}-v_{B}$$

being ν*_B_* = ω*_B_*/*k_B_*.

**Figure 4.**
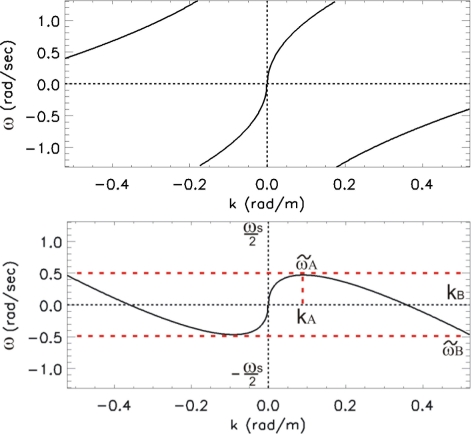
*Upper panel*: folded spectrum as Figure 2, referred to progressive waves, before of compensating the aliasing effect. *Lower panel*: Unfolded spectrum obtained by applying the virtual current *Ũ* of Equation (17) to the spectrum of the upper panel.

When relation (11) holds, the collected data will be affected by aliasing and folded (aliased) shells arise in the second and fourth quadrant; this phenomenon is shown in the upper panel of Figure 4 (the measurement parameters are the same of Table 1).

Accordingly, for a value of the surface current *U* larger than *Û*, the wave spectrum components affected by aliasing are located at *k_m_* *≤|k|≤k_B_* where now *k_m_*, see Figure 3, is given by:

(12) $$\sqrt{\left|k_{m}\right|g}+k_{m}U=\frac{\omega_{s}}{2}$$

leading to:

(13) $$k_{m}=\frac{g+\omega_{s}U-\sqrt{g^{2}+2\omega_{s}\mathrm{Ug}}}{2U^{2}}$$

**Figure 5.**
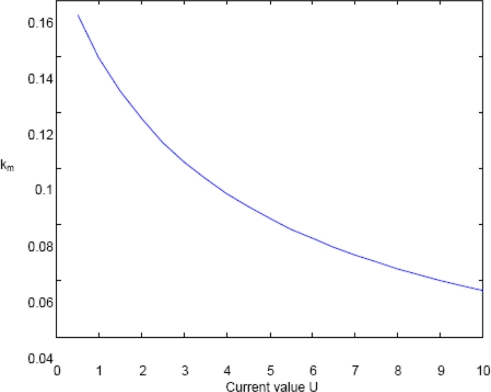
Plot of Equation (13) depicting the decreasing behaviour of the maximum allowable value *k_m_* with respect to the surface current *Û*.

Figure 5 depicts the relation (13) where the allowable wave-number *k_m_*, accounting for the range where the aliasing is not present, is given as function of the sea surface current *U* ; the reader can appreciate how the maximum allowable value *k_m_* behaves as a monotonic decreasing function of the surface current *U*. Finally, it has be noted that when *U* → 0, the estimated value of *k_m_* in (13) is equal to the one specifically evaluated in the case without surface current [compare Equation (13) with Equations (6) and (7)].

## 4. The Strategy to Mitigate the Folding Problem

This Section is devoted at presenting a strategy with the aim of mitigating the effect of the spectrum folding on the sea-state reconstruction results. The proposed strategy moves within the same framework of the techniques presented in [10–12], where the reconstruction of the aliased dispersion shells is achieved by exploiting spectral symmetries. Here, the difference of the proposed approach compared to the ones in [10–12] is concerned with the procedure used to reconstruct the folded (aliased) dispersion shells. In particular, the here proposed approach can be summarized as made up of three main steps.

The *first step* begins with the accurate determination of the sea surface current by means of the approach proposed in [18]; such a sea surface current estimation approach has shown good rejection performances, even against data affected by the aliasing. The knowledge of the sea surface current is exploited for the evaluation of the folding point defined through Equations (12) and (13); after, a “virtual surface current” is applied with the aim of turning from the folded spectrum to a non folded spectrum.

The *second step*, is the same adopted in the approach described in [10–12] and is concerned with the exploitation of a zero-padding technique.

This second step is preparatory to the *third step*, that consists of the passage from the zero padded/assembled spectrum to the original one by “erasing the virtual surface current”.

As said above, the first step aims at assembling the sea wave spectrum in the allowable Nyquist band [−*ω_s_*/2,*ω_s_*/2]. Such an assembling procedure is achieved by exploiting a *“virtual negative surface current U_v_”* in order to consider the *“virtual dispersion relation* *ω̃*(*k*)*”* expressed by:

(14) $$\tilde{\omega }(k,\tilde{U})=\sqrt{\left|k\right|g}+\mathrm{kU}-{\mathrm{kU}}_{v}=\sqrt{\left|k\right|g}-k\tilde{U}$$

where *Ũ* = *U_v_* − *U.*

In particular, the choice of the surface virtual current *U_v_* is performed with the aim of minimizing the quantity *G*(*Ũ*) = max_*k*∈[0,*k_B_*]_|*ω̃*(*k*,*Ũ*)| that defines the bandwidth of the assembled spectrum. From a mathematical point of view, the virtual current *U_v_* has to be chosen as the one that globally minimizes the cost function *G*(*Ũ*) = max_*k*∈[0,*k_B_*]_|*ω̃*(*k*,*Ũ*)|.

**Figure 6.**
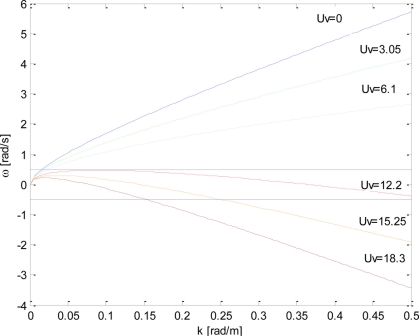
Behaviour of *ω̃(k*,*Ũ*) (according to Equation(14)) for a fixed value of the surface current U = 7 m/s and for different values of the virtual surface current U_v_ = [0,3.05, 6.1, 12.2, 15.25, 18.3] m/s.

With the aim of addressing the problem of the global minimization of *G*(*Ũ*), we investigate the behavior of *ω̃*(*k*,*Ũ*) [see Equation (14)] for different values of the virtual current; Figure 6 depicts the behavior of *ω̃*(*k*,*Ũ*) for several values U_v_ = [0,3.05, 6.1, 12.2, 15.25, 18.3] m/s.

For small values of the virtual current *U_v_* (see the green and gray lines in Figure 6, corresponding to values of U_v_ equal to 3.05 and 6.1 m/s, respectively), the function *ω̃*(*k*) behaves as a monotically increasing function in the interval [0,*k_B_*] and the application of the negative virtual current −*U_v_* entails the beneficial effect of the decrease in the extent of the allowable bandwidth given by:

(15) $${\tilde{\omega }}_{A}={\text{max}}_{k\in [0,\mathrm{kB}]}|\tilde{\omega }(k,\tilde{U})|={\tilde{\omega }}_{\text{max}}=\tilde{\omega }\left(k_{B},\tilde{U}\right)=\sqrt{\left|k_{B}\right|g}-k_{B}\tilde{U}=\sqrt{\left|k_{B}\right|g}-k_{B}\tilde{U}$$

**Figure 7.**
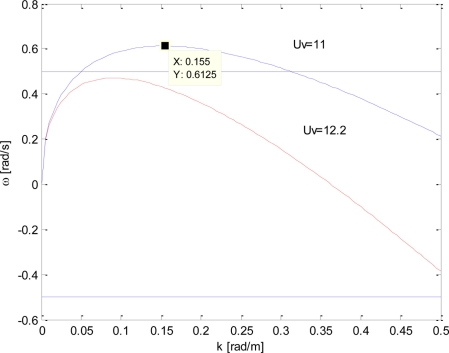
Behaviour of *ω̃(k, Ũ*) (according to Equation (14)) for a fixed value of the surface current U = 7 m/s and for the two different values of the virtual surface current U_v_ = [11, 12.2] m/s. The dotted point accounts for the maximum point (*k*_max_, *ω*_max_).

The behavior mentioned above (*ω̃*(*k*) monotically increasing function of the range [0,*k_B_*]) does not hold when the values of the current *Ũ* increases so that $k_{\text{max}}=\frac{g}{4{\tilde{U}}^{2}}\leq k_{B}$, being *k*_max_ the value where the function *ω̃*(*k*) attains its maximum, *i.e.*, $\tilde{\omega }\left(k_{\text{max}}\right)={\tilde{\omega }}_{\text{max}}=\frac{g}{4\tilde{U}}$. This situation is exemplified by the blue line of Figure 7, where a virtual surface current U_v_ equal to 11 m/s is used.

From the considerations above, it seems that it could be possible to increase *Ũ* with the result that the bandwidth extent ${\tilde{\omega }}_{A}=\omega_{\text{max}}=\frac{g}{4\tilde{U}}$ decreases. However, this statement does not hold for further increasing values of the current *Ũ*; in fact, at the increase in the virtual current, the function *ω̃*(*k*) assumes also negative values whose maximum absolute value is attained at *k_B_* (see curves at U_v_ = 12.2 m/s, 15.25 m/s and U_v_ = 18.3 m/s of Figure 6).

By exploiting the above considerations about the behavior of the function *ω̃*(*k,Ũ*), we perform the choice of the *U_v_* so to achieve minimum bandwidth condition given by:

(16) $${\tilde{\omega }}_{A}=\tilde{\omega }\left(k_{\text{max}}\right)=-{\tilde{\omega }}_{B}=-\tilde{\omega }\left(k_{B}\right)$$

(this choice corresponds to the curve at U_v_ =12.2 m/s in Figure 6).

The solution of the Equation (16) in the unknown *Ũ* provides the solution:

(17) $$\tilde{U}=\frac{\sqrt{k_{B}g}+\sqrt{2k_{B}g}}{2k_{B}}=\frac{1+\sqrt{2}}{2}v_{B}$$

**Figure 8.**
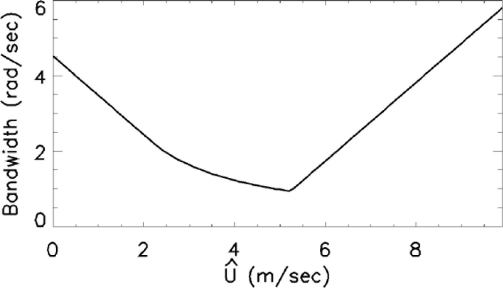
Behavior of the spectral bandwidth *G*(*Ũ*).

The choice of the virtual current according to (17) is numerically justified from Figure 8 that depicts the function *G*(*Ũ*) = max_*k*∈[0,*k*_*b*_]_|*ω̃*(*k,Ũ*)| as function of the looked for current value *Ũ*. The value where the function *G*(*Ũ*) attains the minimum is equal to the one provided by Equation (17) and states the goodness of the choice in (16) and (17).

Let us turn now to exploit the above reasoning to set-up the de-aliasing strategy. First of all, we note that, for the values of the current *Ũ* given by Equation (17), the bandwidth is equal to ${\tilde{\omega }}_{A}=\tilde{\omega }\left(k_{\text{max}}\right)={\tilde{\omega }}_{\text{max}}=\frac{g}{4\tilde{U}}=\frac{\sqrt{gk_{B}}}{2(1+\sqrt{2})}$; such a relation permits to state that the bandwidth is dependent only on the maximum value *k_B_* of the wave-number that one is interested to investigate.

The aim of the proposed mitigation strategy is to exploit the virtual surface current so to constrain the unfolded spectrum in the allowable band $\left[-\frac{\omega_{s}}{2},\frac{\omega_{s}}{2}\right]$ (dictated by the time step Δ*t* used to sample the radar image).

The lower panel of Figure 4 shows the overall result of the change of variable applied to the folded spectrum (reported in the upper panel of the same Figure) as result of the ‘application’ of the virtual current chosen on the basis of Equation (17).

In this way, we can state that the unfolding spectrum technique is successfully applied when the inequality ${\tilde{\omega }}_{A}=\frac{\sqrt{gk_{B}}}{2(1+\sqrt{2})}\leq \frac{\omega_{s}}{2}$ holds; this inequality entails that the spectrum is reliably unfolded for the range of the wave-number $k\in \left[0,\;{\left(\frac{\omega_{s}\left(1+\sqrt{2}\right)}{\sqrt{g}}\right)}^{2}\right]$. Accordingly, as long as the value of the Nyquist frequency *ω_s_* decreases, the reliable wave-number range, where the unfolding of the spectrum is reliably performed, decreases. The value $k_{B}={\left(\frac{\omega_{s}\left(1+\sqrt{2}\right)}{\sqrt{g}}\right)}^{2}$ permits to define the maximum bandwidth within which it is possible to obtain the unfolding of the spectrum; in fact, by substituting $k_{B=}{\left(\frac{\omega_{s}\left(1+\sqrt{2}\right)}{\sqrt{g}}\right)}^{2}$ in the relation (8) and after simple manipulations, we obtain the value of the maximum bandwidth within which it is possible to obtain the unfolding of the spectrum as:

(18) $$\omega_{\mathrm{sN}}=\omega_{s}\;(1+\sqrt{2})+{\left(\frac{\omega_{s}\;\left(1+\sqrt{2}\right)}{\sqrt{g}}\right)}^{2}\;U$$

Once the change of variables in (14) has been performed, a zero padding on the image spectrum *F*(*ω̃*(*k*),*k*) can be applied in order to get the new sampling frequency:

(19) $$\omega_{\mathrm{sN}}=N\omega_{s}\;\text{with}\;N\geq \mathrm{round}\left(\frac{\omega_{\mathrm{sN}}}{\omega_{s}}\right)$$

**Figure 9.**
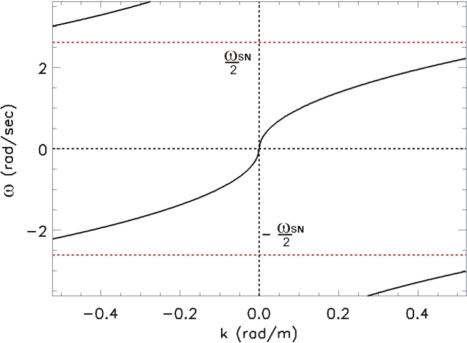
Zero padded non-folded spectrum *F_N_*(*ω(k)*,*k*) obtained form the spectrum of the upper panel of Figure 4 by applying the proposed strategy to solve the aliasing phenomenon.

The final step consists in the addition, by means of a change of variables, of the (inverted) virtual surface current *U_v_* to the X band radar images in order to shift the zero padded unfolded spectrum to the original one (see Figure 9):

(20) $$F_{N}\;\left(\tilde{\omega }(k),k\right)\to F_{N}\;\left(\omega (k),k\right)$$

where the the pedix N indicates the zero padded spectrum and $\omega (k)=\tilde{\omega }(k)+{\mathrm{kU}}_{v}=\sqrt{\left|k\right|g}+\mathrm{kU}$.

As highlighted in Figure 9, the image spectrum *F_N_* (*ω*(*k*),*k*) is not affected by the aliasing phenomenon. Accordingly, it may be then filtered and inverted by using the procedure already proposed in Section 2 with the final aim of estimating the sea surface elevation *η*(*x,t*).

## 5. Numerical Results

**Figure 10.**
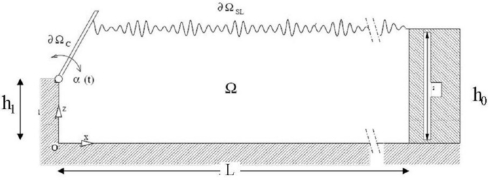
Pictorial sketch of the mathematical problem. The geometrical parameters are h_0_ = 3.6 m, h1 = 1.8 m, L = 230 m.

This Section aims at presenting results conforming the effectiveness of the strategy described in Section 4. To this end, synthetic data, generated by a fully 2D numerical wave-maker [20] reproducing the physical conditions existing in a real wave tank (see Figure 10) will be used. The spatial and time evolution of a wave train propagating on the free-surface ∂Ω*_SL_* and generated through a hinged paddle ∂Ω*_c_* moving with angular velocity *a*(*t*), has been investigated by a non viscid model. In this framework the mathematical statement is described through the Laplace equation for the velocity potential function.

Once the velocity potential is computed on the boundary domain, the nonlinear free-surface equations are stepped forward by a fourth-order Runge-Kutta scheme and the motion of the wavemaker is updated.

A domain decomposition technique has been used [21], with the aim to save computational time and memory effort for the simulation of long time wave evolution. Further details of the numerical model used as well as of the treatment of the free-surface can be found in [20].

A JONSWAP sea spectrum with H1/3 = 0.094 m and T0 = 1.97 s has been simulated in the numerical wave tank; a scale factor of 20 has been used to reconstruct the wave elevations corresponding to the full scale sea state (H1/3 = 1.88 m and T0 = 8.8 s). Here, H1/3 represents the significant sea surface elevation, and T0 the modal period associated with the prescribed spectrum. The JONSWAP sea spectrum is used in this paper, but the proposed approach is also suitable for other kinds of sea spectra.

The sea-state data have been generated by performing an average of the spectra of three sea states with the duration of 6 m. The data are sampled with a time-step of 0.34 s and a spatial step of 0.6 m. In particular, a total number of Nx = 6,306 spatial samples has been used leading to an extent of 3,750 m. For the time discretization Nt = 1,066 time-samples have been considered by achieving an overall acquisition time of 6 min.

The averaged data has been decimated; in fact, the samples actually used to perform the reconstruction are Nx = 630 and Nt = 32 with a step of Δx = 5.9 m and Δt = 2.4 s, according to the spectral parameters of Table 1. This leads to a spatial and time extent of 3,717 m and 76.8 s (in full scale), respectively. A surface current *U* of 7 m/s and directed (head waves) as the wave motion has been added to the data.

The corresponding radar data have been generated by exploiting the procedure proposed in [2], where the model of the electromagnetic scattering exploits the geometrical optics approximation and the shadowing and tilt modulation are accounted for. In particular, we simulated the electromagnetic scattering in the case of a X-band radar radiating at 9.3 GHz and located at the height of 20 m with respect to the sea zero-level.

**Figure 11.**
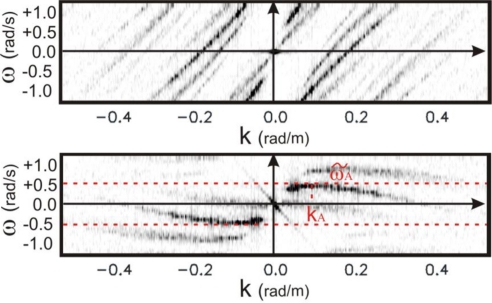
Upper panel: folded image spectrum *F*(*ω(k*),*k*). Lower panel: not folded spectrum *F*(*ω̃(k)*,*k*) after the variable change of Equation (14).

The upper panel of Figure 11 shows the image spectrum of the data affected by the folding phenomenon: the quantity ω_B_ (see Table 1) does not satisfy Equation (5). Clearer shells in Figure 11 represent the higher modes of the image spectrum due to modulation phenomena generated during the radar image formation [1].

The change of variable in (14) is performed by exploiting the value of the virtual current m/s U_v_ = 12.2m/s m/s that allows us to achieve the value *Ũ* = 5.2 m/s according to Equation (17); this allows us to achieve the unfolded spectrum as shown in the lower panel of Figure 11.

**Figure 12.**
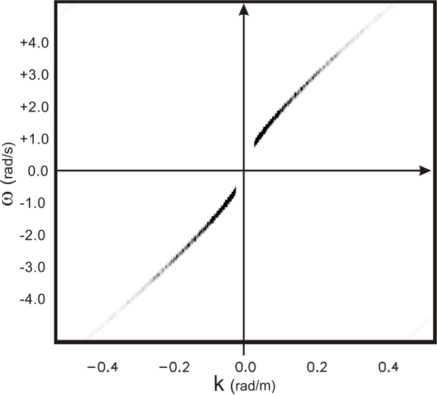
Wave spectrum *F_N_*(*ω(k)*,*k*) after the MTF and filtering procedure shown in the block diagram of Figure 1.

**Figure 13.**
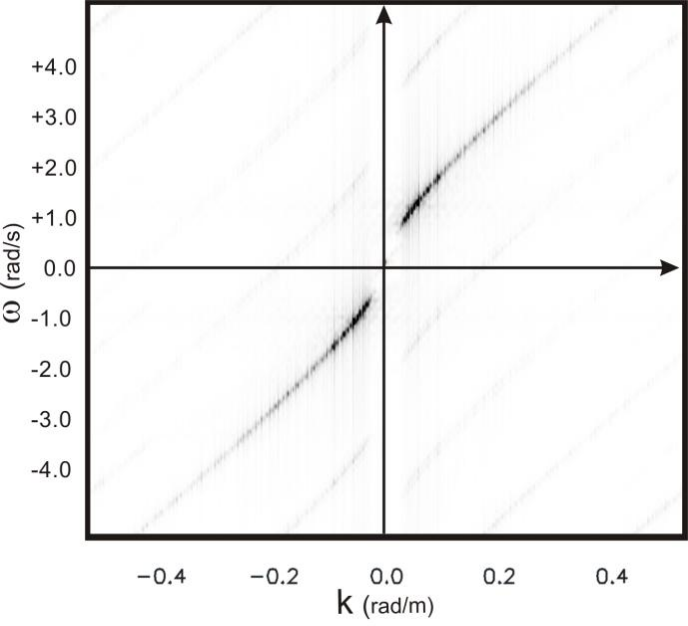
True (non-folded) sea-wave spectrum sampled with correct space and time steps.

Figure 12 depicts the sea-wave spectrum after use of the filtering procedure described in the block diagram of Figure 1; the result is in good agreement with the reference (unfolded) sea-wave spectrum (see Figure 13) obtained from the same synthetic data at the finest spatial and time resolution (Nx = 6,306 spatial samples and Nt = 1,066 time-samples).

**Figure 14.**
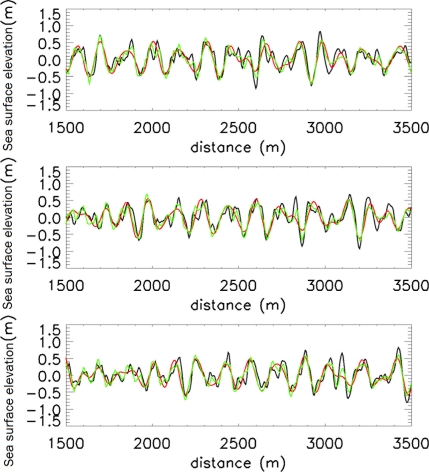
Reconstructed function *η_NA_*(*x,t*) (green line) and *η_A_*(*x,t*) (red line), at the time instant [0, 36, 76] s, compared to the simulated sea surface elevation *η(x,t)* (black line).

**Figure 15.**
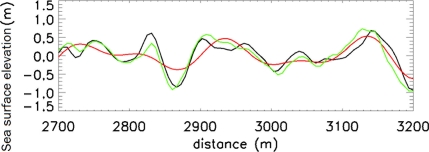
Zoom of middle panel (t = 36 s) of Figure 13 in the spatial range of [2,700, 3,200] m.

As further assessment of the effectiveness of the proposed method, the panels of Figure 14 depict the comparison among the reconstructed sea surface elevation functions *η_NA_*(*x,t*) (achieved by using the present strategy, green line), *η_NA_*(*x,t*) (reconstructed without the use of any de-aliasing strategy, red line) and the true (simulated) sea surface elevation *η(x,t)* at the time instants [0, 36, 76] s, respectively. A zoom of the middle panel of Figure 14 for the spatial range [2,700, 3,200] m is shown in Figure 15.

In order to give a quantitative evaluation of the effectiveness of the proposed strategy the normalized quadratic norm error:

(21) $$E_{Y}=\mathrm{mean}\left\{\frac{\sum_{i}{\left|\eta (x,t)-\eta_{Y}(x,t)\right|}^{2}}{\sum_{i}{\left|\eta (x,t)\right|}^{2}}\right\}$$

is calculated for the reconstructed signals *η_NA_*(*x,t*)(Y = NA) and *η_A_*(*x,t*) (Y = A), respectively. Equation 20 provided *E_NA_* = 0.44 and *E_A_* = 0.62 which correspond to an improvement of 30% in the quality of the reconstruction.

Finally, we present a comparison between the proposed procedure and the strategy implemented according to the scheme in [12] exploiting a “shifting” procedure based on spectral symmetries. In particular, we assume to make an error of 3.5 m/s in the knowledge of the sea surface current so that we assume erroneously that *U* = 3.5 m/s.

**Figure 16.**
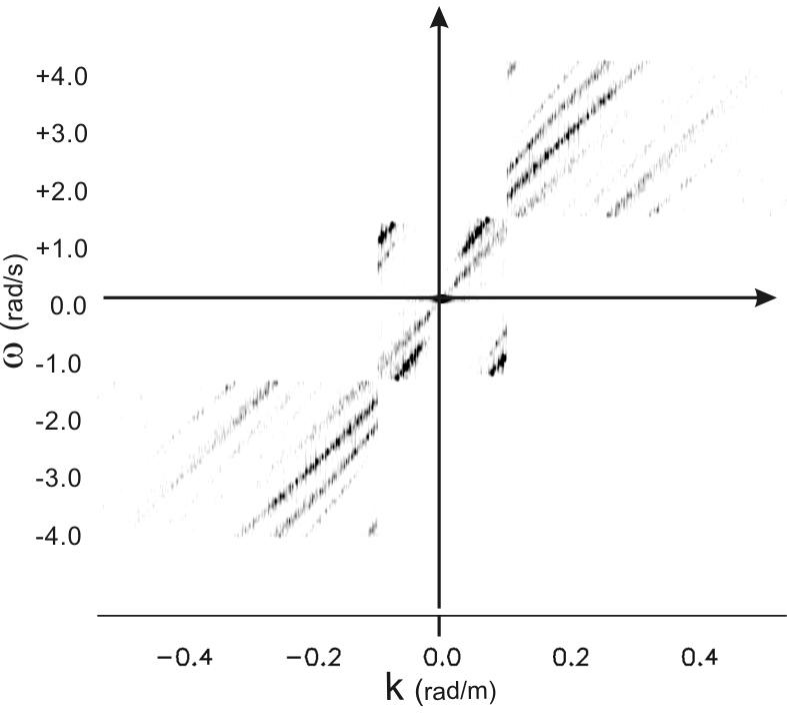
The assembled spectrum according to the procedure in [12].

Figure 16 depicts the re-constructed spectrum according to the procedure outlined in [12]; it can be noted that in this case, due to the fact that the performances of the strategy is strongly dependent on the accurate estimation of the sea surface current, the spectrum is not well reconstructed and its support exhibits undesired “jumps”.

**Figure 17.**
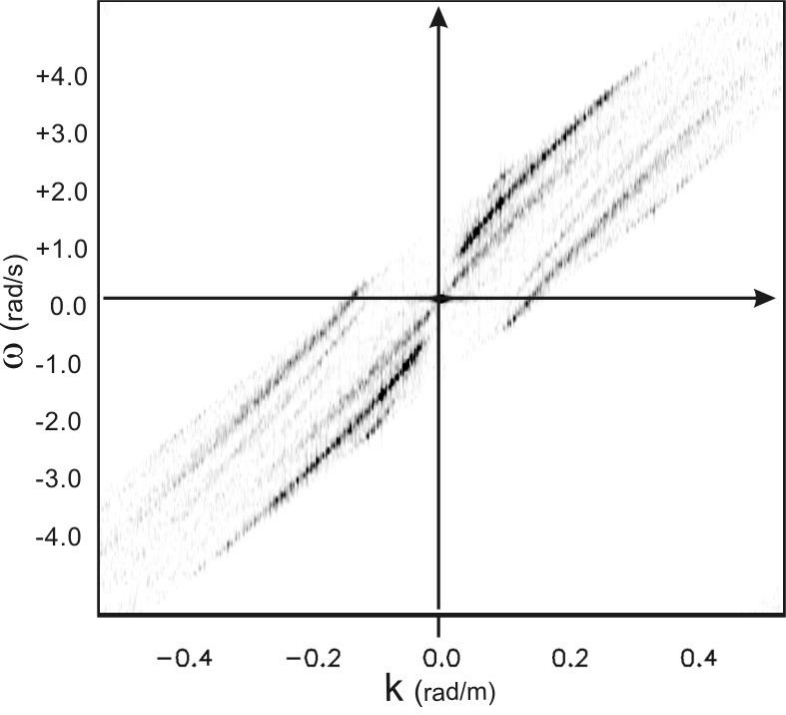
The assembled spectrum according to the procedure proposed herein.

Figure 17 depicts the spectrum assembled by means of the proposed strategy. It shows how, even in presence of inaccurate knowledge of the sea surface current, it is possible to assemble the spectrum so as to avoid jumps so that the support of the spectrum behaves as a continuous function *ω(k)* At this point, by using this unfolded spectrum, it is simpler to re-evaluate the sea surface current and after to reconstruction of the sea surface elevation is possible.

## 6. Conclusions

This paper has dealt with the question of the aliasing problem that may arise in the reconstruction of the sea surface elevation starting from the images collected by a X-band radar system. In particular, the effect of a non appropriate time-step, adopted in radar data acquisition, has been thoroughly analyzed for the progressive waves. The investigation was concerned with both the cases of the absence and presence of the surface current and we have shown, by simple theoretical considerations, how the effect of an increasing value of the surface current badly affects the aliasing problem.

Based on these theoretical considerations, a strategy was proposed to mitigate the aliasing problem and its effectiveness has been shown by an analysis of synthetic data. The proposed method has been presented for unimodal sea states. As future development of this research activity, more challenging cases where the aliasing problem occurs, such as regressive and mixed waves, should be addressed and where the further question of the ambiguities arises [12]. This investigation would permit applying the method in an operational way for different sea state conditions. Finally, as future activity, we will address the operational capability of the proposed technique to the more realistic 3D case and in the real world by processing experimental data.

## Figures and Tables

**Table 1.** Parameters for generation of the folded spectrum.

**Parameter**	**Value**
Sampling frequency (*k_s_*)	1.05 rad/meter
Sampling frequency (*ω_s_*)	2.61 rad/s
Frequency step (Δ*k*)	1.7E-3 rad/meter
Frequency step (Δ*ω*)	0.08 rad/s
Bandwidth (*k_B_*)	0.52 rad/meter
Bandwidth (*ω_B_*)	2.3 rad/s
